# An Effective Screening Method and a Reliable Screening Trait for Salt Tolerance of *Brassica napus* at the Germination Stage

**DOI:** 10.3389/fpls.2019.00530

**Published:** 2019-04-26

**Authors:** Hui Wu, Jianrong Guo, Chengfeng Wang, Kailun Li, Xiaowen Zhang, Zhen Yang, Maoteng Li, Baoshan Wang

**Affiliations:** ^1^Shandong Provincial Key Laboratory of Plant Stress, College of Life Sciences, Shandong Normal University, Ji’nan, China; ^2^Shandong Provincial Key Laboratory of Microbial Engineering, School of Biologic Engineering, Qilu University of Technology (Shandong Academy of Sciences), Ji’nan, China; ^3^Department of Biotechnology, College of Life Science and Technology, Huazhong University of Science and Technology, Wuhan, China

**Keywords:** *Brassica napus* L., germination, evaluation, screening of salt-tolerant index, salt tolerance

## Abstract

Salinity is a major and complex abiotic stress that inhibits plant growth and reduces crop yield. Given the global increase in soil salinity, there is a need to develop salt-tolerant species. *Brassica napus* L. is an important oilseed crop with some level of salt tolerance. However, few studies have evaluated its salt tolerance thoroughly or screened for traits that can be reliably evaluated for salt tolerance. Here, we evaluated salt tolerance in 549 *B. napus* inbred lines with different genetic backgrounds using the membership function value (MFV) of certain traits, including the germination rate, root and shoot length, root and shoot fresh weight, and total fresh weight. According to the evaluation criteria-mean MFV, 50 highly salt-tolerant, 115 salt-tolerant, 71 moderately salt-tolerant, 202 salt-sensitive, and 111 highly salt-sensitive inbred lines were screened at the germination stage. We also developed a mathematical evaluation model and identified that the salt tolerance index of shoot fresh weight is a single trait that reliably represents the salt tolerance of *B. napus* germplasm at the germination stage. These results are useful for evaluating and breeding salt-tolerant *B. napus* germplasm.

## Introduction

Salinity is a major abiotic stress that inhibits plant growth and reduces crop yield (Kumar et al., 2010; Tavakkoli et al., 2011). About 800 million hectares of farmland are affected by salinization worldwide (Cramer et al., 2011). In China, there are about 100 million hectares of salinized land, and this number is predicted to increase (Song and Wang, 2015; Yang and Wang, 2015). Saline soil is mainly caused by poor irrigation practices, along with saline groundwater in inland regions and high ocean tides in the coastal regions (Ganie et al., 2016).

The ability of the plant to survive and complete its life cycle under saline conditions is dependent on its salt tolerance, which varies among different species and growth stages (Zeng et al., 2002; Akbari et al., 2007). Thus, the best way to use saline soil is to screen for and develop salt-tolerant crop species and varieties (Ghoulam and Fares, 2001; Ashraf et al., 2012). Plant response to salinity is mainly reflected in morphological, physiological, biochemical, and molecular changes. For example, salinity stress results in osmotic stress, ion toxicity, and nutritional imbalances (Jones and Gorham, 2002), which reduces growth and alters the levels of cell metabolites (Rhodes et al., 2002).

Seed germination is the first stage of the plant’s life cycle, and is negatively affected by salinity (Azza et al., 2007; Feizi et al., 2007). Several previous studies have shown that seed germination is extremely sensitive to salinity in most plant species (Heenan et al., 1988). Abbas et al. (2013) demonstrated that the percent germination, shoot and root length, and dry weight of rice (*Oryza sativa*) were reduced with increasing levels of NaCl. Similarly, in dicotyledonous cabbage (*Brassica oleracea*), seed germination and the growth of roots and buds were also inhibited under salt stress (Jamil et al., 2007). Therefore, salt tolerance at the germination stage is critical for the successful growth of plants in saline conditions.

Oilseed rape (*Brassica napus*) is an important salt-tolerant oilseed crop (Maas, 1993; Ashraf and McNeilly, 2004). Determining the reliable index and traits is important for salt-tolerant breeding. Many studies have shown that halophytes, such as *Suaeda salsa* and *Salicornia europaea*, have high salt tolerance at the germination stage though their germination rates are also reduced (Song et al., 2008; Hakim et al., 2010; Deng et al., 2014; Guo et al., 2018), while all crops are highly sensitive to salinity (Carpycy et al., 2009; Hakim et al., 2010; Cokkızgın, 2012; Ding et al., 2018). However, the indicators for evaluation of salt tolerance at germination stage are not consistent between different species. Various screening methods for salinity tolerance have been developed, including plant growth (Sayed, 1985), germination rate, leaf or root elongation (Garcia et al., 1995), K^+^/Na^+^ discrimination (Asch et al., 2000), and Cl^−^ exclusion (Rogers and Noble, 1992). The Na^+^ content in the shoot is considered a reliable trait of salt tolerance in barley (*Hordeum vulgare*; Pakniyat et al., 1997). In rice, Makihara et al. (2001) proposed that the photosynthetic rate of excised leaf blades is a good indicator of salt tolerance. However, few studies have focused on salt tolerance in *B. napus*. According to Long et al. (2013), root and shoot length can be used as an early indicator for evaluating salt tolerance of *B. napus*. Hu et al. (2018) evaluated the salt tolerance of a small number of *B. napus* germplasms using a comprehensive analysis of multi-index results, but an effective screening indicator was not presented. Thus, a reliable screening trait for salt tolerance of *B. napus* and an effective large-scale screening method at the seed germination stage have yet to be determined.

In this study, 549 *B. napus* germplasms (inbred lines) were used to evaluate salt tolerance. In addition, we established a mathematical evaluation model and found a reliable screening trait for investigating the salt tolerance of *B. napus* at the germination stage. These results provide a basis for breeding salt-tolerant *B. napus.*

## Materials and Methods

### Plant Materials

A total of 564 inbred lines seeds of *B. napus* with different genetic backgrounds were harvested in 2016 and stored in a refrigerator at temperatures < 4°C prior for germination experiments. These seeds were kindly provided by Professor Maoteng Li from Huazhong University of Science and Technology.

### Determination of Optimal Salt Stress Concentration

Fifteen inbred lines were randomly selected from the 564 inbred lines and used to determine the optimal salt concentration within a concentration of 50, 100, 150, 200, and 250 mmol L^−1^ NaCl. Seeds treated with distilled water (0 mmol L^−1^ NaCl) were assigned as the control. Seeds were sterilized with 70% alcohol for 15 min, washed five times with distilled water, and then soaked in distilled water for 12 h. Twelve uniform and healthy seeds were selected from each of the 15 inbred lines and germinated in 9-cm Petri dishes lined with a double-layer of blotting paper and containing 9 mL of NaCl solution at the concentrations stated above. The seeds were cultured in a growth chamber at 28 ± 3°C/23 ± 3°C (day/night) with a relative humidity of 70%, and a light intensity of 600 μmol m^−2^ s^−1^ (14 h light/10 h dark). Seeds were considered to have germinated when the radicle length was ≥2 mm. It was considered as the optimum stress concentration of NaCl at which the salt-injury index was 50% of the control.

### Screening of Salt-Tolerant Inbred Lines

The 9 cm diameter Petri dishes were divided into three equal parts, with each part containing 12 uniform seeds of a *B. napus* inbred line and treated with the optimum NaCl concentration and distilled water (control), respectively, and there were three biological replicates in each treatment. For each inbred line, 36 seeds were in each treatment (control and 200 mmol L^−1^ NaCl).

### Determination of Physiological Parameters

The number of germinated seeds was recorded every day for 7 days. The fresh weight and seedlings length were also measured at 7 days after sowing (DAS).

To evaluate the salt tolerance of *B. napus* inbred lines at the germination stage, the germination rate, the fresh weight of the shoot and root (mg) and the shoot and root length (cm) were determined. These were calculated using the formulae below.

Germination rate (GR): Germination rate was calculated 7 DAS:

$$\text{GR}={\text{G}}_{7}/\text{T}\times 100\%$$

**Table 1:** Correlation analysis between salt tolerance indices of shoot fresh weight (STI of SFW), root fresh weight (STI of RFW), shoot length (STI of SL), root length (STI of RL), and total fresh weight (STI of TFW) of 438 *B. napus* inbred lines in the presence of 200 mmol L^−1^ NaCl.

		STI of SFW	STI of RFW	STI of SL	STI of RL	STI of TFW
STI of SFW	Pearson correlation	1	0.660^∗∗^	0.930^∗∗^	0.855^∗∗^	0.912^∗∗^
STI of RFW	Pearson correlation	0.660^∗∗^	1	0.621^∗∗^	0.624^∗∗^	0.724^∗∗^
STI of SL	Pearson correlation	0.930^∗∗^	0.621^∗∗^	1	0.859^∗∗^	0.844^∗∗^
STI of RL	Pearson correlation	0.855^∗∗^	0.624^∗∗^	0.859^∗∗^	1	0.817^∗∗^
STI of TFW	Pearson correlation	0.912^∗∗^	0.724^∗∗^	0.844^∗∗^	0.817^∗∗^	1

*^∗∗^Correlation is significant at the 0.01 level (two-tailed).*

Where G_7_ is the number of germinated seeds on the 7 DAS, T is the total number of seeds (Alvarado et al., 1987; Ruan et al., 2002).

In order to reduce the effect of the germination rate (faster or slower) on the later growth of root and shoot, the abnormal seedlings (particular faster or slower) were removed during the germination and uniform seedlings were used to compare the following parameters at 7 DAS.

Shoot length (SL) and root length (RL) were measured individually at 7 DAS.

Shoot fresh weight (SFW) and root fresh weight (RFW) were determined for each replication at 7 DAS.

Total fresh weight (TFW) is the sum of SFW and RFW of an individual plant.

The salt-tolerance index (STI) is the ratio of the value for the NaCl-treated plant/value for the control.

Salt-injury index (SII): SII = 1-STI.

### Salt Tolerance Evaluation

The salt tolerance of *B. napus* was evaluated using the membership function value (MFV) using the fuzzy comprehensive evaluation method (Chen et al., 2012). The MFV of salt tolerance was calculated using the following equation:

$${\text{X}}_{\text{i}}=(\text{X}-{\text{X}}_{\min})/({\text{X}}_{\max}-{\text{X}}_{\min})\times 100\%$$

where, X_i_ is the MFV of STI in a specific inbred line, X is the actual measured value of STI in a specific inbred line, and X_max_ and X_min_ are the maximum and minimum values observed in all inbred lines, respectively (Ding et al., 2018). According to the average value of the MFVs of each trait, the salt tolerance of the inbred line was evaluated. The MFVs of all inbred lines ranged from 0 to 1.

For each genotype, mean MFV is the average of MFVs of germination rate, shoot weight, root weight, shoot length, root length, and total fresh weight. So, each genotype has its own mean MFV, the bigger the mean MFV, the higher the salt tolerance.

#### Hierarchical Cluster Analysis

hierarchical cluster analysis was also used to evaluate salt tolerance. The salt tolerance was divided into five levels: highly salt tolerant (HST), salt tolerant (ST), moderately salt tolerant (MST), salt sensitive (SS), highly salt sensitive (HSS).

Using the software of SPSS to perform multiple regression analysis on mean MFV (dependent variable Y) and STI value (independent variable Xi) for each genotype. A mathematical evaluation model for salt tolerance was established: Y = β_1_X_1_+ β_2_X_2_+ β_3_X_3_+ β_4_X_4_+ β_5_X_5_+ μ, where Y is the mean MFV, X_1_ is the STI of SFW, X_2_ is the STI of RFW, X_3_ is the STI of SL, X_4_ is the STI of RL, X_5_ is the STI of TFW, β is the B of unstandardized coefficient, and μ is constant. Constant (μ) means the random error term.

### Statistical Analysis

The data are presented as means ± standard deviation (SD). The values were analyzed using SPSS (version 13.0) for windows and ANOVA followed by Tukey’s *post hoc* test. All tests were performed using SPSS Version 13.0 for Windows (SPSS, Chicago, IL, United States).

## Results

### Determination of Optimal Salt Concentration

**FIGURE 1:**
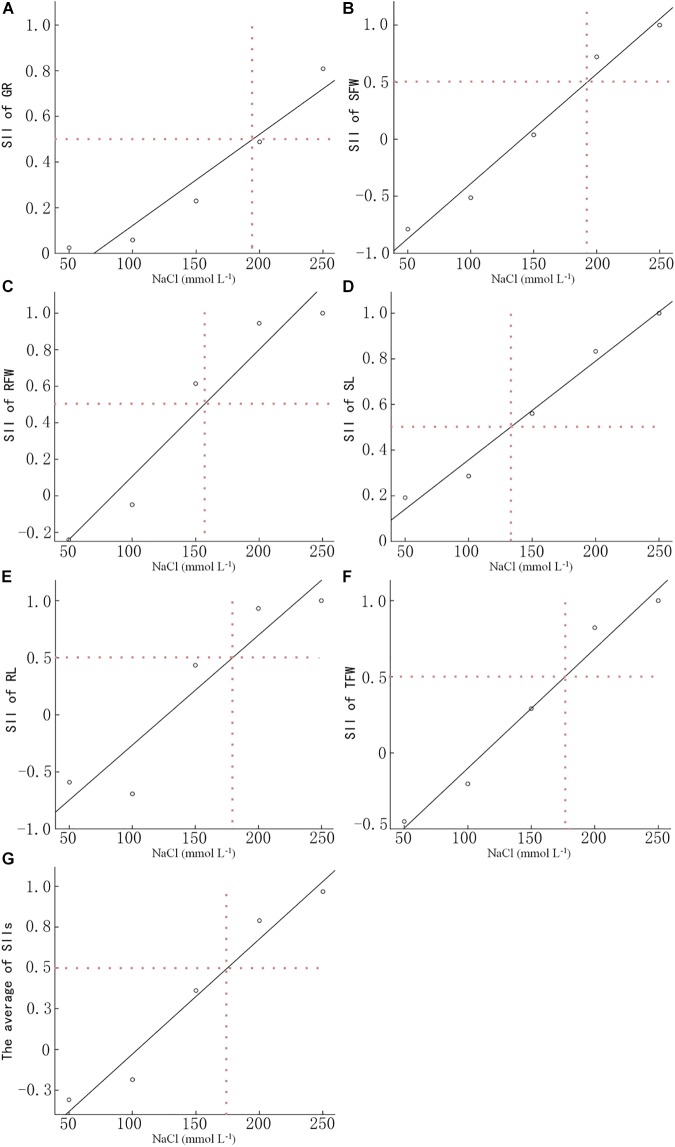
Determination of the optimal NaCl concentration for evaluating salt tolerance. The NaCl concentration of the salt-injury index is 0.5 of the germination rate **(A)**, shoot fresh weight **(B)**, root fresh weight **(C)**, shoot length **(D)**, root length **(E)**, total fresh weight **(F)**, and the average **(G)** of 15 *Brassica napus* inbred lines under different NaCl concentrations. Data in the figure are means of 15 *B. napus* inbred lines for each indicator under each concentration of NaCl.

We determined the GR, SFW, RFW, SL, RL, and TFW of 15 *B. napus* inbred lines at 7 DAS (Supplementary Table S1). The SII of each indicator was calculated based on the data in Supplementary Tables S1, S2 and the average SII for each indicator over all 15 lines was analyzed using linear regression analysis (Figure 1). When treated with 191.3 mmol L^−1^ NaCl, the SII of the GR decreased to 50% of control. The NaCl concentration at which the SII of SFW decreased to 50% was 191.5 mmol L^−1^. For RFW, SL, RL, and TFW, the concentration of NaCl which led to a 50% decrease in the SII was 156.1, 133.2, 175.7, and 175.9 mmol L^−1^, respectively. The average concentration of these four NaCl concentrations was 173.9 mmol L^−1^. Therefore, 200 mmol L^−1^ NaCl was used in the present study to evaluate the salt tolerance of the other 549 *B. napus* inbred lines.

**Table 2:** Multiple regression analysis for salt tolerance indices of shoot fresh weight (STI of SFW), root fresh weight (STI of RFW), shoot length (STI of SL), root length (STI of RL), and total fresh weight (STI of TFW) in the presence of 200 mmol L^−1^ NaCl.

Model		Unstandardized coefficients		Standardized coefficients	*t*	Significance
		μ or B	*SE*	β		
1	Constant	0.045	0.003		17.594	0
	STI of SFW	0.257	0.020	0.365	12.956	0
	STI of RFW	0.234	0.026	0.103	9.060	0
	STI of SL	0.257	0.024	0.245	10.786	0
	STI of RL	1.064	0.085	0.205	12.524	0
	STI of TFW	0.131	0.02	0.140	6.668	0

*Dependent variable: mean MFV, P < 0.01. Where B is unstandardized coefficients (β). Constant (μ) means the random error term.*

**Table 3:** Salt tolerance verification of multiple regression analysis with their MFVs.

No. of inbred lines	STI of SW	STI of RW	STI of SL	STI of RL	STI of TFW	Mean MFV	Y
							
67	0.939	0.192	0.276	0.104	0.726	0.622	0.608
127	0.662	0.206	0.496	0.092	0.536	0.595	0.560
335	0.781	0.152	0.441	0.138	0.599	0.638	0.620
73	0.759	0.099	0.292	0.073	0.620	0.541	0.497
343	0.605	0.118	0.459	0.079	0.442	0.517	0.488
567	0.585	0.146	0.436	0.092	0.466	0.534	0.501
6	0.019	0.619	0.248	0.035	0.181	0.401	0.319
85	0.407	0.028	0.294	0.044	0.298	0.378	0.318
564	0.431	0.071	0.420	0.068	0.339	0.414	0.397
52	0.000	0.000	0.000	0.000	0.000	0.143	0.045
269	0.000	0.000	0.000	0.000	0.000	0.105	0.045
436	0.000	0.000	0.000	0.000	0.000	0.141	0.045

*Where numbers in the first column refer to No. of inbred lines; salt tolerance index of shoot fresh weight (STI of SFW), root fresh weight (STI of RFW), shoot length (STI of SL), root length (STI of RL), and total fresh weight (STI of TFW), Y = 0.045 + 0.257^∗^STI of SFW + 0.234^∗^STI of RFW + 0.257^∗^STI of SL + 1.064^∗^STI of RL + 0.131^∗^STI of TFW.*

### Correlation Analysis of Physiological Parameters Under Salt Stress

The GR, SFW, RFW, SL, RL, and TFW of each inbred line were measured at 200 mmol L^−1^ NaCl at 7 DAS (Supplementary Table S3), and the STI of each indicator was calculated (Supplementary Table S4; data from the un-germinated inbred lines under these conditions is not shown). To determine the relationship (if any) between these physiological parameters under NaCl stress, a correlation analysis was performed (Table 1). There was a positive correlation between any two STIs of SFW, RFW, SL, RL, and TFW, and the highest correlation coefficient (0.930) was between the STI of SFW and the STI of SL. The second-highest correlation coefficient was that between the STI of SFW and the STI of TFW (0.912), and the lowest correlation coefficient was between the STI of RFW and the STI of SL (0.621).

### Salt Tolerance Evaluation

**FIGURE 2:**
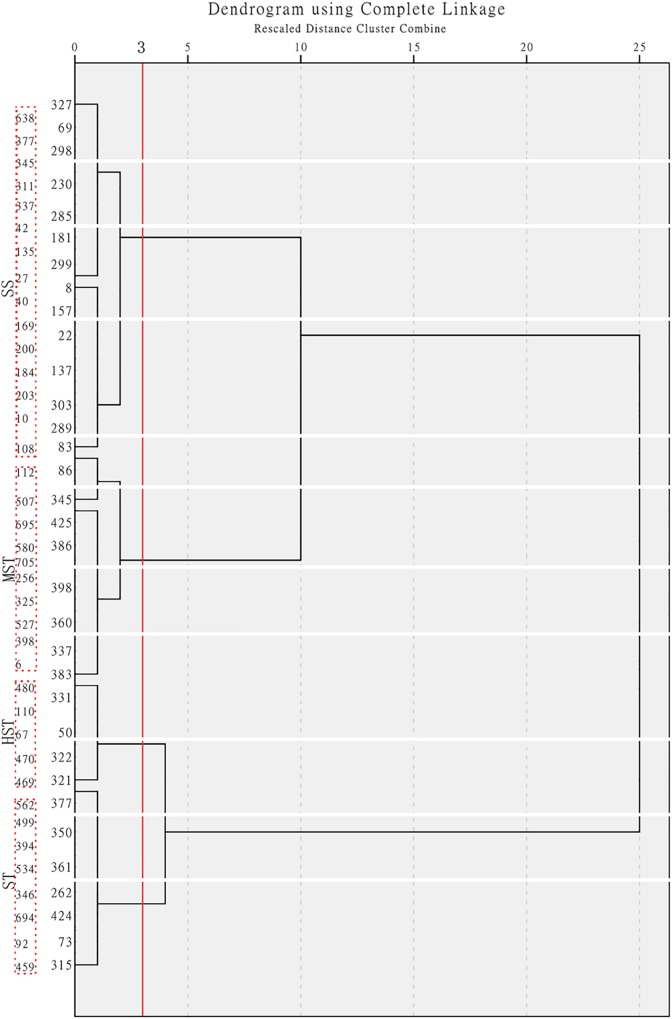
Hierarchical cluster analysis based on the Furthest Neighbor to evaluate the salt tolerance of 438 *B. napus* inbred lines. HST, high salt tolerance; ST, salt tolerance; MST, moderately salt tolerant; and SS, salt sensitive.

**FIGURE 3:**
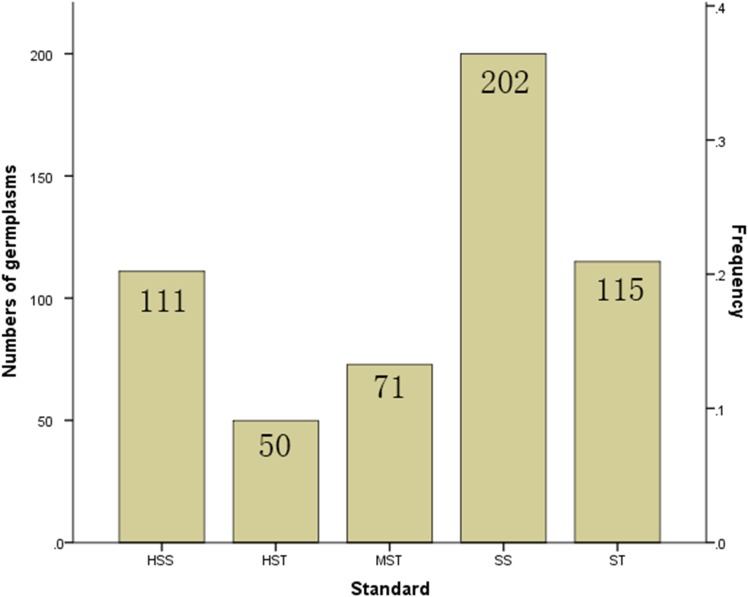
Proportion of the 549 *B. napus* inbred lines with different salt tolerances. HST, highly salt tolerant; ST, salt tolerant; MST, moderately salt tolerant; SS, salt sensitive; HSS, highly salt sensitive based on hierarchical cluster analysis of salt tolerance.

The MFV of each indicator and mean MFV was calculated (Supplementary Table S5), and a hierarchical cluster analysis based on the Furthest Neighbor was used to evaluate the salt tolerance of *B. napus* inbred lines (Figure 2). The salt tolerance of 438 *B. napus* inbred lines was divided into four levels with a distance of three between each level: HST, ST, MST, and SS. In addition, the un-germinated *B. napus* inbred lines in the experiment were classified as HSS. The difference in salt tolerance (distinguished by the MFV of each indicator after germination) between the *B. napus* inbred lines is shown in Supplementary Table S6. Among all the *B. napus* inbred lines analyzed, 50 were classified as HST, 115 as ST, 71 as MST, 202 as SS, and 111 as HSS (Figure 3).

### Establishment of a Salt Tolerance Evaluation Model and Screening for a Reliable Single Indicator

A mathematical evaluation model for analyzing the salt tolerance of 438 *B. napus* inbred lines was established using multiple regression analysis. The unstandardized coefficients of the STI of SFW, RFW, SL, RL, and TFW were 0.257, 0.234, 0.257, 1.064, and 0.131, respectively. The random error term was 0.045. Therefore Y = 0.045+0.257^∗^STI of SFW+0.234^∗^STI of RFW+0.257^∗^STI of SL+1.064^∗^STI of RL+0.131^∗^STI of TFW (*P* < 0.01) (Table 2), where Y represents the salt tolerance of a *B. napus* inbred line.

To test whether the mathematical evaluation model can predict the salt tolerance of any inbred line, three inbred lines in each of the different clusters were randomly selected and their Y values were calculated (Table 3). The results indicated that the formula can be used to evaluate the salt tolerance of any *B. napus* inbred line at the germination stage. For example, the Y of # 67 (HST) is 0.045+0.257^∗^0.939+0.234^∗^0.192+0.257^∗^0.276+1.064^∗^0.104+ 0.131^∗^0.726; therefore Y = 0.608, and its mean MFV is 0.622; the Y of # 73 (ST) is 0.497, and its mean MFV is 0.541; and the Y of # 564 (MST) is 0.397, and its mean MFV is 0.414. The values of mean MFV and Y were very close. In addition, we use this model to analyze salt tolerance that the genotypes used for determination of optimal salt concentration. The results of salt tolerance analysis are shown in Supplementary Table S6. We find that the higher salt tolerance (Y value) of the genotypes which showed higher growth under mild salt stress as compared to those of controls. Therefore, our model is reliable and salt tolerance can be predicted by calculating the Y value of any *B. napus* inbred line using the STIs of growth parameters such as SFW, RFW, SL, RL, and TFW at the germination stage.

**FIGURE 4:**
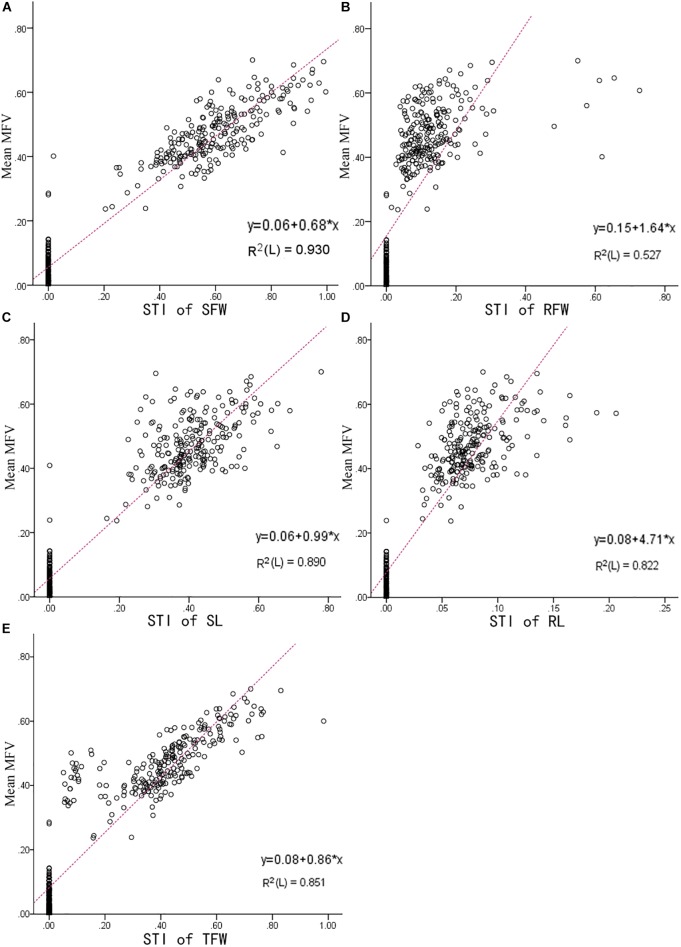
The linear fit between the STI of each indicator and the mean MFV of an individual *B. napus* inbred line (438 *B. napus* inbred lines with different tolerances to salt stress). **(A)** Is between Mean MFV and STI of SFW (salt tolerance index of shoot fresh weight); **(B)** is between Mean MFV and STI of RFW (salt tolerance index of root fresh weight); **(C)** is between Mean MFV and STI of SL (salt tolerance index of shoot length); **(D)** is between Mean MFV and STI of RL (salt tolerance index of root length); **(E)** is between Mean MFV and STI of TFW (salt tolerance index of total fresh weight).

The higher the mean MFV, the higher the salt tolerance (Ding et al., 2018). In our study, the mean MFV was affected by the STI of SFW, RFW, SL, RL, and TFW, which means that the higher STI value of each indicator, the higher the MFV value. To determine which indicator is most reliable in reflecting salt tolerance, a linear model between the STI of each indicator and the mean of MFV was fitted. As shown in Figure 4, the R^2^ between the mean MFV and the STI of SFW was the highest (0.930), the R^2^ between the mean MFVs and the STI of SL, RL, and TFW were slightly lower, i.e., 0.89, 0.822, and 0.851, respectively, and the R^2^ between the mean MFV and the STI of RFW was the lowest (0.527). These results are consistent with the results presented in Table 2, in which the standardized beta coefficient between the mean MFV and the STI of SFW was also the highest. Overall, our results suggest that shoot fresh weight can be used as a reliable trait to evaluate the salt tolerance of *B. napus* inbred lines at the germination stage.

**FIGURE 5:**
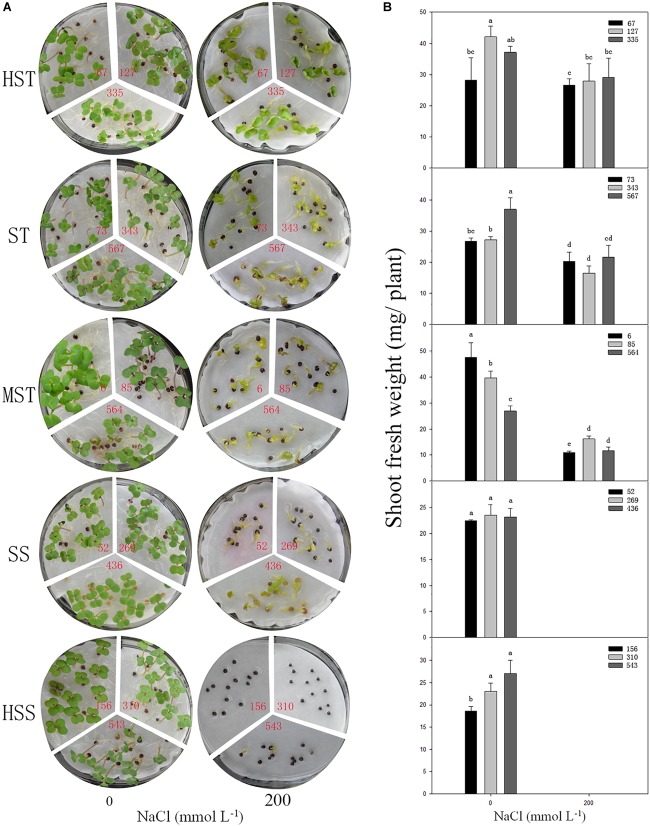
Phenotypes **(A)** and shoot fresh weight **(B)** of the seedlings of different salt-tolerant *B. napus* inbred lines at 7 DAS under 200 mmol L^−1^. No. of HST (highly salt tolerant): 67, 127, and 335; ST (salt tolerant): 73, 343, and 567; MST (moderately salt tolerant): 6, 85, and 564; SS (salt sensitive): 52, 269, and 436; and HSS (highly salt sensitive): 156, 310, and 543 *B. napus* lines. Data are means (*n* = 18) ± SD. Different letters indicate significant difference at *P* < 0.05.

To test if the shoot fresh weight reflects salt tolerance accurately, three inbred lines were randomly selected, from each salt-resistant category and the phenotypes of the seedlings, and the shoot fresh weights were determined at 7 DAS in 200 mmol L^−1^ NaCl (Figure 5). There was no significant difference (*P* > 0.05) in growth between the different *B. napus* inbred lines in the control (no NaCl) treatment. However, the growth and shoot fresh weight were significantly reduced by NaCl as the salt tolerance of the *B. napus* inbred lines decreased (HSS > SS > MST > ST > HST). When treated with 200 mmol L^−1^ NaCl, the leaves of the HST *B. napus* inbred lines were still green, while the leaves of the ST *B. napus* inbred lines turned yellow. The HSS *B. napus* inbred lines barely germinated in 200 mmol L^−1^ NaCl. The average shoot fresh weight of an individual plant was 27.82 mg (HST lines) and 12.89 mg (MST lines). Furthermore, the total fresh weight of SS *B. napus* lines was close to 0 g (Figure 5B).

## Discussion

Most plants have developed salt-resistant stress mechanisms, but their ability to resist salt stress varies widely among different species and cultivars (Ashraf and Wu, 1994). According to Maas (1993), *B. napus* has a certain level of salt tolerance. However, the salt concentration that *B. napus* can tolerate is much lower than that of a true halophyte (Deng et al., 2014; Yuan et al., 2016; Guo et al., 2018). When *B. napus* is cultivated in saline soil, growth is inhibited and seed oil production is low. In this study, 200 mmol L^−1^ NaCl was determined as the optimal concentration to evaluate the salt tolerance based on the salt resistance of 15 *B. napus* inbred lines using a concentration gradient experiment because germination rate of *B. napus* inbred lines were differently reduced (Supplementary Table S1). Subsequently, the salt resistance of 549 *B. napus* inbred lines with different genetic backgrounds was determined under 200 mM NaCl. Interestingly, some of the genotypes showed higher root and shoot growth under 50 or 100 mM NaCl as compared to those of controls. *B. napus* is considered to be a crop with a certain salt tolerance (Maas, 1993). For euhalophytes, a moderate salinity significantly promotes vegetable and reproductive growth due to efficient ion compartmentalization and succulence (Song et al., 2008; Qi et al., 2009; Yang et al., 2010; Song and Wang, 2015; Guo et al., 2018). Our previous results showed that halophyte, for example *Thellungilla halophila* is more adaptive to salinity compared with *Arabidopsis thaliana* at stages of seed germination and seedling establishment (Guo et al., 2012). For some *B. napus* genotypes, the reason of the elevated growth at low concentration NaCl treatments may be related to faster and higher water absorption and physiological start than controls. We speculate that these genotypes have higher ion content and lower water potential under mild salt stress as compared to those of controls, which led to a higher root and shoot growth. However, the ion content needs to be determined in the next step to verify this speculation.

We observed that the germination rate of *B. napus* seeds from all cultivars decreased in the presence of 200 mmol L^−1^ NaCl. According to Ayaz et al. (2000), the decline in germination rate is most likely due to metabolic disorders that occur under salt stress conditions. We believe that the germplasms in the 549 *B. napus* inbred lines that did not germinate were HSS. Whether the seed germinates or not is a qualitative change that does not accurately express quantitative changes and therefore does not reflect the plant’s ability to grow under salt stress. Therefore, the salt tolerance of *B. napus* cannot be accurately evaluated using germination rate alone. Hu et al. (2018) evaluated the salt tolerance of 88 *B. napus* cultivars by the MFV using the fuzzy comprehensive evaluation method. Principal component analysis is used for fuzzy evaluation, and determining the contribution rate of the principal components involves subjective factors, which therefore cannot objectively express the salt tolerance of the cultivars themselves. In our study, the MFV of STI of growth parameters such as SFW, RFW, SL, RL, and TFW at the germination stage in a specific inbred line was calculated based on the data of 438 inbred lines respectively. Then, for each genotype, mean MFV (the average of MFVs of germination rate, shoot weight, root weight, shoot length, root length, and total fresh weight) was calculated. The mean MFV is a multiple indicator for evaluating plant salt tolerance, and the bigger the mean MFV, the higher the salt tolerance. Therefore, the mean MFV and a hierarchical cluster analysis based on the Furthest Neighbor was used to evaluate the salt tolerance of *B. napus* inbred lines (Figure 2). The salt tolerance of 438 *B. napus* inbred lines was determined and divided into 50 HST, 115 ST, 71 MST, and 202 SS. In addition, the 111 un-germinated *B. napus* inbred lines were classified as HSS.

When we determine the salt tolerance of one or some *B. napus* genotypes, it is difficult to get an answer without a large number of other genotypes as a comparison. It is also laborious and complicated to evaluate salt tolerance using the MFV. To easily and reliably evaluate salt tolerance of one *B. napus* inbred line or variety, we developed a mathematical formula for the evaluation of salt tolerance using multiple regression analysis. Consequently, we can estimate the salt tolerance of any *B. napus* inbred line by calculating the Y value. A relatively accurate evaluation of salt tolerance can be obtained in our mathematical model. The bigger the Y value, the higher the salt tolerance (Table 3). It is the first time to establish a mathematical evaluation model to predict the salt tolerance of *B. napus* during germination. This mathematical formula will be useful in the screening of salt tolerance of *B. napus*.

According to Long et al. (2013), the root and shoot length can be used as an early indicator for evaluating salt tolerance in *B. napus*. Hu et al. (2018), however, considered that a comprehensive analysis of multi-index results would better reflect the salt tolerance of *B. napus* during germination. However, measuring multiple traits is difficult and time-consuming, especially on a large scale. In this study, based on R^2^ between the mean MFV and multiple indicators, the STI of SFW appears to be a single that reliable screening trait for the assessment of salt tolerance of *B. napus* inbred lines. This result provides the foundation for less difficult and more time-efficient evaluation of salt tolerance, and for salt-tolerant breeding of *B. napus*.

## Conclusion

In this study, we determined the optimal NaCl concentration (200 mmol L^−1^) for the salt tolerance evaluation of *B. napus*. 50 HST, 115 ST, 71 MST, 202 SS, and 111 HSS of *B. napus* inbred lines were screened during the germination stage. Furthermore, we proposed a mathematical evaluation model to assess the salt tolerance. We found that the SFW was a reliable trait for evaluating salt tolerance of *B. napus* inbred lines. These results will greatly contribute to the evaluation and breeding of salt tolerant *B. napus*.

## Author Contributions

BW and ML designed the research. HW, JG, CW, KL, XZ, and ZY performed the experiments. HW and BW wrote the manuscript with contributions from the other authors. All authors analyzed the data.

## Conflict of Interest Statement

The authors declare that the research was conducted in the absence of any commercial or financial relationships that could be construed as a potential conflict of interest.
